# Autolysis in Crustacean Tissues after Death: A Case Study Using the *Procambarus clarkii* Hepatopancreas

**DOI:** 10.1155/2021/2345878

**Published:** 2021-01-12

**Authors:** Xiaoli Huang, Guanqing Xiong, Xia Chen, Ruisi Liu, Minghao Li, Lili Ji, Xiaoli Zhang, Yi Geng, Yangping Ou, Defang Chen, Lizi Yin, Liangyu Li, Shiyong Yang

**Affiliations:** ^1^Department of Aquaculture, College of Animal Science & Technology, Sichuan Agricultural University, Wenjiang Chengdu, 611130 Sichuan, China; ^2^Fishery Research Institute, Chengdu Academy of Agriculture and Forestry Sciences, Wenjiang Chengdu, 611130 Sichuan, China; ^3^Sichuan Key Laboratory of Meat Processing, Chengdu University, Longquanyi Chengdu, 610100 Sichuan, China; ^4^College of Veterinary Medicine, Sichuan Agricultural University, Wenjiang Chengdu, 611130 Sichuan, China

## Abstract

Autolysis is an internal phenomenon following the death of an organism that leads to the degradation of tissues. In order to explore the initial stages of autolysis and attempt to establish reference standards for tissue changes after death, we studied the rapidly autolyzing tissue of the crayfish hepatopancreas. Samples from the hepatopancreas of crayfish were examined 0, 5, 10, 30, 60, and 120 minutes after death. Histological and ultrapathological examinations and evaluations and apoptotic cell counts were conducted to determine the initiation time and degree of autolysis. The results showed that autolysis in the hepatopancreas of crayfish began within 5 minutes. Initially, autolysis manifested in the swelling of hepatic tubular cells and the widening of mesenchyme. Cells undergoing autolysis showed severe organelle necrolysis. Based on these observations, tissue samples should be collected and preserved within five minutes to avoid interfering with histopathological diagnoses.

## 1. Introduction

Histological examination is key to the diagnosis of disease, and as of yet, no comparable alternative examination has been developed. In modern medical research, histology has enabled clinical medicine to observe histological structures and pathological changes, link metabolic and functional changes to diseased organs, explore the etiology and pathogenesis of diseases, and identify the internal and mutual relationships between pathological changes and clinical manifestations [1]. However, poor tissue fixation, nonstandardized preparation of paraffin sections, inaccurate reading of slices, and other methodological problems can seriously impact the diagnosis of histopathology. One particular obstacle that interferes with the diagnosis of etiology is autolysis, which occurs when the dissected tissue is not fixed in a timely manner after operation.

Autolysis is an internal reaction that occurs upon death and can occur spontaneously without external intervention [2]. By as early as 1985, Babayan and Bezrukov had defined the hydrolysis reaction during cell death in which intracellular biopolymers under the action of hydrolases form low molecular weight products as “autolysis” [3]. Further study of autolysis revealed that autolysis in animal body tissues was caused by the activation of enzyme systems in tissues by biological, physical, chemical, and/or other factors after death, so as to spontaneously destroy and degrade the tissue structure, through cell softening or liquefaction, a global and irreversible reaction that occurs in almost all organisms after death [2].

Previous studies have also found the timing of tissue autolysis after death to vary greatly among animals. For example, the liver of Homo sapiens did not begin to dissolve until 12 hours after death [4]. This was similar to the changes in liver tissue of chicken, which began 12 hours after death [5]. However, edema of hepatocytes, as observed under a transmission electron microscope, occurred 1 hour after death while cell necrosis did not occur until 15 hours after death [6]. In aquatic animals, due to the relatively high tissue water content and abundance of hydrolases in their internal organs, the time to autolysis after death is relatively short. The autolysis time of anchovies was not more than 10 hours [7], and tilapia reached its optimum autolysis time within 6 hours. Compared to aquatic vertebrates, crustacean tissues are even more prone to rapid autolysis [8]. In our previous work, we observed that the tissues and organs of shrimp and crab lost their original forms in a short period of time after death, which made tissue sampling especially time sensitive if an accurate pathological diagnosis is to be ensured [9, 10]. However, the exact progression of tissue autolysis in crustaceans after death and how it affects tissue structures are still unknown.

*Procambarus clarkii*, commonly known as “crayfish” and classified under Arthropoda, Malacostraca, Decapoda, *Cambaridae* [11], has been increasingly cultivated in China in recent years. To explore the initiation and progression of autolysis in crayfish cells, hepatopancreases were dissected from multiple crayfish and examined after different time delays. Histological observation, ultrapathological examination, and cytological damage observation were conducted to determine the longest acceptable histological sampling time and establish reference standards for tissue changes due to autolysis.

## 2. Material and Methods

### 2.1. Animals and Chemicals

Adult crayfish (*n* = 54) were provided by the Chengdu Shenghuayuan Agricultural Science and Technology Co. LTD (Chongzhou, Chengdu, China). Their average body weight was 15 ± 1.2 g, and average body length was 8 ± 0.7 cm. The crayfish were acclimated in quarantine for 1 week prior to the initiation of the experiment, at which time their health status was evaluated. Animals were randomly divided into 6 groups.

The eBioscience ™ Annexin V-FITC Apop Kit was purchased from Invitrogen.

### 2.2. Samples

At the beginning of the experiment, all crayfish from all six groups were sacrificed at the same time, and their hepatopancreases were collected by dissection. To avoid the influence of other factors, the carapace was removed first, and then the hepatopancreas was gently removed and placed in the carapace where it remained exposed to the laboratory air (room temperature 25°C). Tissue samples were collected after 0, 5, 10, 30, 60, and 120 minutes from the 6 experimental groups, respectively.

### 2.3. Histological Detection

The hepatopancreas samples of the six groups, collected from at different times after death, were fixed in the Davidson's AFA fixative and processed in paraffin using standard procedures. Hepatopancreas tissues were also trimmed into cassettes, dehydrated in graded ethanol solutions, cleared in xylene, and embedded in paraffin wax. Sections (4 *μ*m thick) for hematoxylin and eosin (H&E) staining were prepared prior to microscopic analysis (CX 33, Olympus, Japan).

### 2.4. Transmission Electron Microscopy (TEM) Examination

Hepatopancreas tissues were prepared for TEM by soaking in 2.5% glutaraldehyde for 2-4 hours, followed by washing 3 times with 0.1 M phosphate buffer (pH = 7.4) for 15 min. Samples were dehydrated in series concentration (50%, 70%, 80%, 90%, 95%, 100%, and 100%) of ethanol for 15 min each, then dehydrated twice in 100% acetone for 15 min. After being infiltrated overnight, samples were embedded for 48 h at 60°C and sectioned into 60-80 nm slices. Samples were then stained with uranyl acetate and lead citrate. Images were acquired using a transmission electron microscopy (HT7700, HITACHI, China).

### 2.5. Assessment of Pathological Changes

The degree of hepatic tubular necrosis, hepatic tubular atrophy, hepatic tubule epithelial cells floating away, R cell necrosis and fusion, interstitial swelling, and hepatic tubular lumen deformation were graded according to the scoring system proposed by Baums and colleagues [12]. Histologic changes were given a score (S) ranging from 0 to 6, depending on their degree and extent: (0) no change; (2) mild; (4) moderate; and (6) severe. Intermediate values could also be assessed.

Reference histopathological scoring standards ranging from 0 to 6 points on the same scoring system were used to assess the severity of the ultrapathological changes. For each ultrathin section, 5 visual fields (VF) were selected for image collection. Then, a blind evaluation was conducted for the ultrapathological scoring. Finally, scores of images in each ultrathin section were categorized using identifiers.

### 2.6. Hepatopancreas Cell Apoptosis Measurement

For measurement of cell apoptosis, three crayfish hepatopancreases were collected 0 and 120 minutes after death. The hepatopancreases were then sampled to determine the percentage of apoptotic cells using flow cytometry. Upon collection, the hepatopancreases were immediately minced to form a cell suspension and filtered through a 300-mesh nylon screen. Cells were washed twice with cold PBS, and the cell pellet was resuspended to make a concentration of 1 × 10^6^ cells/mL in PBS. Then, 5 *μ*L of Annexin V-fluorescein isothiocyanate (V-FITC) (BD Pharmingen, Franklin Lakes, New Jersey, USA) and 10 *μ*L of propidium iodide (PI) (BD Pharmingen) were sequentially added into the 200 *μ*L cell suspension. The cells were then incubated with Annexin V-FITC/PI in the dark for 15 min at room temperature. The cell suspensions were examined for apoptotic cells by the CytoFLEX flow cytometry. The data were analyzed using the Kaluza 2.1 software.

### 2.7. Statistical Analyses

The results are expressed as means ± standard deviations. The results of the histopathological assessments were expressed as the medians and ranges. The significant differences were identified using analysis of variance (ANOVA). The influence of each indicator was tested with one-way analysis of variance and a *t*-test. A value of *P* < 0.05 was considered significant.

## 3. Results

### 3.1. Morphological Changes of the Hepatopancreases

**Figure 1:**
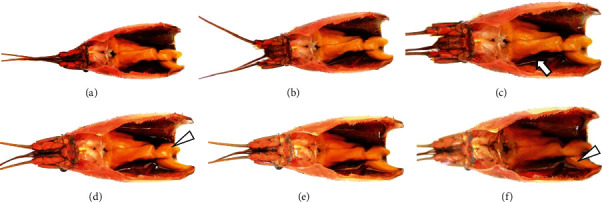
The morphological changes of hepatopancreases at different time point after dissection. (a–f) 0, 5, 10, 30, 60, and 120 min after dissection. Arrows indicate fluid exudation (⟶), and arrowheads show hepatopancreas dissolution (*Δ*).

With increases in time, hepatopancreases showed a trend of dissolution, with the surface becoming gradually more tarnished and the color changing from yellow to dark yellow (Figure 1). At 0 min after the death, hepatopancreas tubules were arranged clearly and neatly, with uniform textures and bright surfaces. No significant change was observed when the hepatopancreases were dissected after 5 minutes, but after 10 minutes, there was a small amount of fluid exudation around the hepatopancreases. Furthermore, the hepatic tubules were slightly dissolved and mixed with exudated fluid at 30 minutes. After 60 minutes, fluid exudation had significantly increased, the hepatic tubular dissolution was more obvious, and the surface color of the hepatopancreases begun to dim, At 120 minutes, the original shape of the hepatopancreases was completely unrecognizable, large amounts of the samples had liquified, and the whole hepatopancreatic structure appeared to be dissolving.

### 3.2. Gradual Autolysis of Hepatopancreases with Time

**Figure 2:**
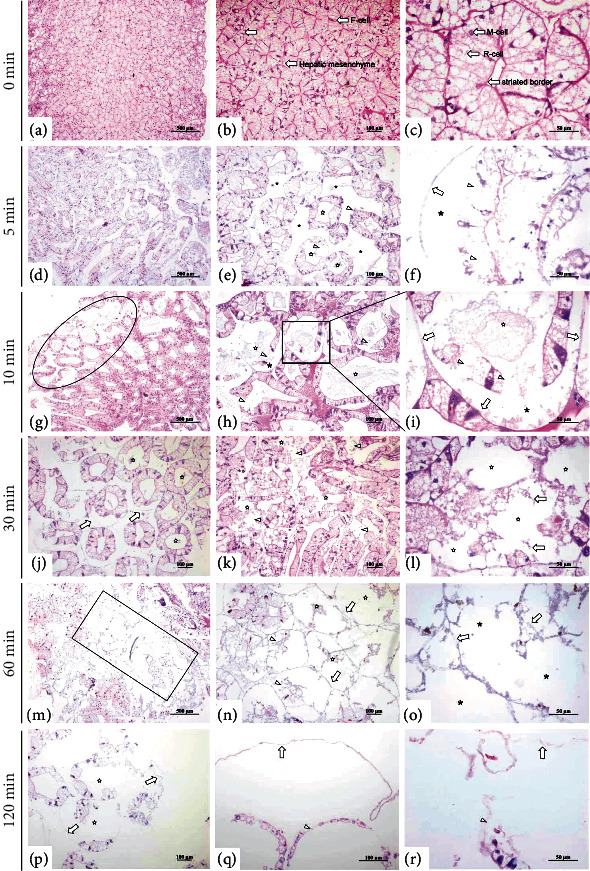
The histological changes of hepatopancreases over time after death. (a–c) Normal hepatopancreatic histological appearance. (d–f) The hepatic tubules were swollen, hepatopancreatic stroma widened (∗), the lumen dilatated (☆), R cell fusion (*Δ*), and hepatic tubule epithelial cells sloughed from the basement membrane (⟶). (g–i) Large areas of mesenchyme expanded at lower power, necrosis of some epithelial cells (∗), accumulation of debris in the lumen (☆), R cells fusion, and necrosis (*Δ*). (j–r) Hepatic tubule epithelial cells sloughed from the basement membrane (⟶); hepatic tubule cell necrosis (☆), and large necrosis areas (∗).

The histological changes of the hepatopancreases increased with time after death, and necrosis of hepatic tubules was the main symptom of autolysis. Under the microscope, the hepatopancreas histology was normal in the 0 min group (Figures 2(a)–2(c)). The hepatopancreases in this group were composed of numerous well-organized hepatic tubules with homogeneous stroma (containing hemolymph) between the tubules and contained stellate cavities with a uniform striate border. The hepatic tubules were mainly composed of hepatopancreas cells, including E cells (embryonic cells), R cells (absorptive cells), F cells (fiber cells), and B cells (secretory cells). However, in the hepatopancreases examined 5 minutes after the death (Figures 2(d)–2(f)), the hepatic tubule mesenchyme had widened slightly, the lumen had also expanded, and the shape of hepatic tubule cavities changed from star-shaped to irregular, and some of the epithelial cells of the hepatic tubules had sloughed off. At 10 minutes after death (Figures 2(g)–2(i)), the lumen had become significantly larger and rounder. In addition, necrosis of the hepatic tubule cells had begun, and debris from the necrotic cells had accumulated in the lumen. When examined 30 minutes after death (Figures 2(j)–2(l)), most of the hepatic tubule lumen had become round, and a large number of hepatic epithelial cells had sloughed off of the hepatic tubules. At high magnification, the necrosis appeared aggravated, which was expressed in lipid droplets fusion and rupture necrosis in R cells. Interestingly, F cells showed minimal necrosis, while B cells showed no significant changes. At 60 minutes after death (Figures 2(m)–2(o)), necrosis was obvious in large areas of the hepatopancreases, some hepatic tubules were thoroughly necrotic, and some hepatic tubules had completely dissolved. In summary, in the first stages of autolysis, the hepatic tubules of the hepatopancreases swelled and the interstitial gaps widened, then necrosis of hepatic cells began, and with increasing time after death, the structure of hepatic tubules eventually disappeared. In addition, we found that different parts of the hepatopancreas underwent different degrees of autolysis in the same sampling time, with generally slower progression in the marginal region and faster in the central region. This might be due to the abundance of enzymes in the central region.

### 3.3. Evaluation of the Histopathological Characteristic of Autolysis

**Figure 3:**
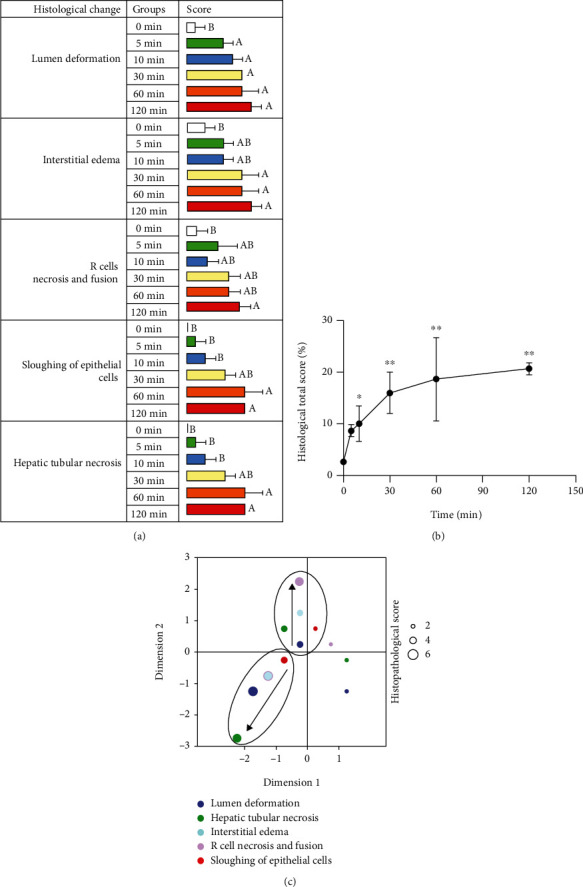
Histopathological scores of crayfish hepatopancreases at different times after death. (a) Histopathological score table. (b) Histopathological total scores. (c) Histopathological scores multiple correspondence analysis.

According to the histopathological scoring of the hepatopancreases (Figure 3(a)), the hepatopancreases changed significantly during autolysis, with hepatic tubular necrosis, hepatic tubular atrophy, sloughing of hepatic tubule epithelial cells, R cell necrosis and fusion, interstitial edema, and hepatic tubular lumen deformation. The pathological changes showed gradual necrotic aggravation from 5 to 60 minutes. In addition, according to the trends of the total scores (Figure 3(b)), histological changes due to autolysis progressed rapidly in the first 30 minutes, but after 30 minutes, the slope of the trend line gradually decreased and finally approached parallel to the *X* axis. According to the multiple correspondence analysis, it was found that the early stage of autolysis was characterized by cellular edema, gradually progressing to cell necrosis as time progressed (Figure 3(c)).

### 3.4. Ultrapathological Changes of Hepatopancreases

**Figure 4:**
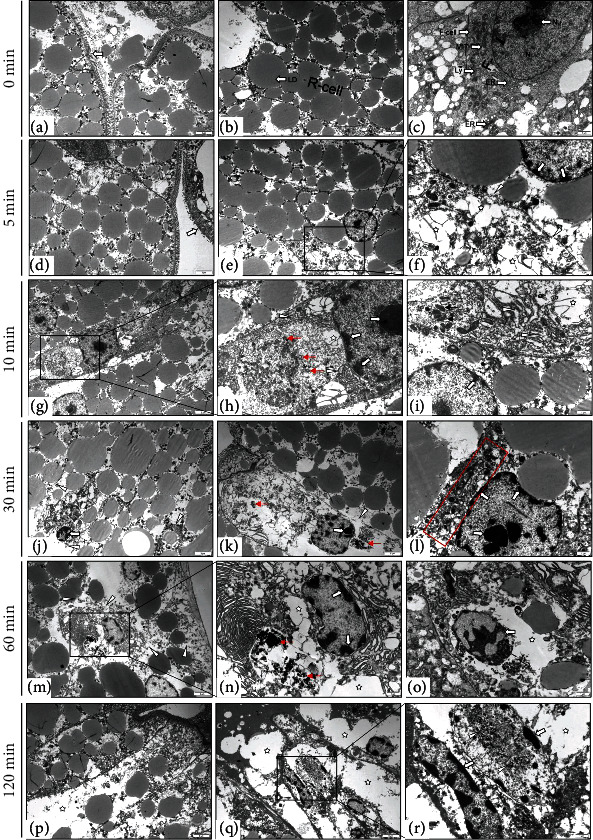
Ultrapathological changes of crayfish hepatopancreas at different times after death. (a–c) The normal hepatopancreas of crayfish was usually composed of R cells and F cells, R cells contained a large number of lipid droplets, and F cells were rich in organelles. (d–f) Microvilli sloughed (white ⟶), R cells ruptured (☆). (g–l) Lysosomes increased (red ⟶), mitochondrial cristae disappeared (black ⟶), endoplasmic reticulum expanded (*Δ*), chromatin condensed, and chromatin border shifted (white ⟶). (m–o) Cell membrane ruptured (*Δ*), and lysosomes released phagocytic substances (red ⟶). (p–r) Cell vacuolation (☆), nuclear necrosis (*Δ*). Nucleus (N), nucleolus (Nu), endoplasmic reticulum (ER), lysosome (Ly), lipid droplet (LD), mitochondrion (mit), and ribosome (RI).

Histological observation showed that necrosis was the main driver of the autolysis process, forming lesions in the hepatopancreases. Therefore, ultrapathological examination of the hepatopancreases was conducted to explore the process of cell necrosis during autolysis. The results showed that severe nuclear necrosis and organelle changes occurred in the hepatopancreas cells during autolysis. At 0 minutes after death (Figures 4(a)–4(c)), the structures of hepatic tubular cells were relatively complete, the microvilli at the edge of the hepatic tubules were neatly organized, the organelles were abundant, and a large number of lipid droplets were present in R cells. However, 5 minutes after death (Figures 4(d)–4(f)), the microvilli had begun to fall off, and a few R cells had become necrotic. High-magnification images revealed that the cell membranes of the necrotic R cells ruptured, nucleus chromatin had condensed and its chromatin border had shifted, the endoplasmic reticulum had expanded, and the mitochondrial cristae were lost. After 10 minutes (Figures 4(g)–4(i)), organelles of R cells decreased, and lipid droplets began to fuse and degrade, and at high magnification, the endoplasmic reticulum appeared severely expanded, and the mitochondrial cristae had disappeared. In addition, while endoplasmic reticulum of F cells had expanded, their nucleus underwent no obvious change. When dead for 30 minutes (Figures 4(j)–4(l)), F cells showed severe necrosis, with ruptured cell membranes, and damage to the two-layer membrane structure of the nucleus. Large vacuoles appeared in the cytoplasm, organelle abundance was significantly reduced, and the endoplasmic reticulum expanded to the point of disappearing. When the sampling time was extended to 60 minutes after death (Figures 4(m)–4(o)), large areas of microvilli of the hepatic tubules had been exfoliated, with severe necrosis of R cells and F cells. Under high magnification, it could be seen that a large number of lysosomes had been released from the cytoplasm. At 120 minutes after death (Figures 4(p)–4(r)), the hepatic tubule cells had been severely necrotized, and most organelles had disappeared from the R cells. In conclusion, complete cell autolysis progressed from early cell vacuolization to the expansion of the endoplasmic reticulum, from condensed nucleus chromatin and a shifted border, to the disappearance of mitochondrial cristae, and from a great reduction in organelles, to ruptured cell membranes, releasing a large number of lysosomes.

The ultrastructural pathological scores were mainly affected by changes in the hepatic tubular cells, especially the R and F cells, which manifested in cytoplasmic vacuolization, ER dilation, chromatin pyknosis, and border shift. In addition, membranolysis, lysosome increases, nuclear shrinkage, and fragmentation were also observed in the hepatopancreases during autolysis (Table 1).

### 3.5. Relationship between Autolysis and Apoptosis

**Figure 5:**
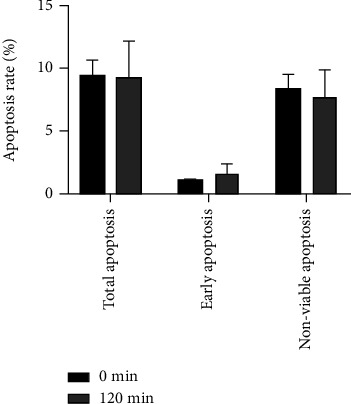
Percentage change of apoptotic cells in hepatopancreas tissues.

Necrosis of hepatopancreas cells was the primary change observed during autolysis, and chromatin solidification and edging were observed in the ultrastructural pathology, both of which are phenomena similar to those that occur during apoptosis. In order to confirm whether the autolysis process was dominated by apoptosis, apoptosis in samples collected at 0 and 120 minutes after death was measured (Figure 5). The results showed that there was no significant difference in the number of apoptotic cells between 0 and 120 minutes samples. Therefore, hepatopancreas autolysis was not driven by apoptosis.

## 4. Discussion

The progression of autolysis in tissues is species specific and depends on the individual organs and the environment in which the body dies, but all tissue sample should be collected in a timely manner. The present study focused on rapidly autolyzing tissue, the crayfish hepatopancreas, to determine the maximum acceptable histological sampling time and attempt to establish reference standards for tissue changes due to autolysis. The histology results showed that under normal temperature conditions, the hepatopancreas tissues started to undergo autolysis within the first 5 minutes after death. Autolysis initially manifested in the swelling of the whole tissue samples, and histological and ultrastructural examination showed that changes became more pronounced over time after death, showing organelle dissolution, endoplasmic reticulum expansion, and the nucleus chromatin pyknosis. Therefore, for this species, it is necessary to collect and preserve samples immediately, within 5 minutes, to avoid confounding the histopathological diagnosis with symptoms of autolysis. In addition, because enzyme activity and gene expressions can also be altered during autolysis, samples should be immediately frozen for molecular analyses following the collection of histological tissue samples.

Not only can autolysis affect pathological diagnosis, but where food safety is concerned, autolysis will affect the taste and integrity of many foods, potentially producing toxic substances that could harm human health. However, autolysis reactions may also prove useful in food processing, as it is the spontaneous self-degradation by organisms and may significantly reduce the cost of enzyme preparation in food processing [13, 14]. One such scenario was true for *Penaeus vannamei*, where autolysis improved the extraction rate of fat from the tissues [15]. Therefore, in food processing, it may be beneficial to explore the molecular mechanisms of autolysis and identify key factors and target genes in the process of autolysis, so as to make rational use of naturally occurring enzymes by targeted inhibition or promotion.

## Figures and Tables

**Table 1:** Ultrapathology scores of hepatopancreases over time.

Ultrapathological change	Groups	Score	Ultrapathological change	Groups	Score
Cytoplasmic vacuolization	0 min	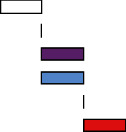	Membronolysis	0 min	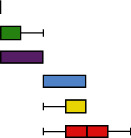
	5 min			5 min	
	10 min			10 min	
	30 min			30 min	
	60 min			60 min	
	120 min			120 min	
ER dilation	0 min	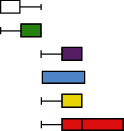	Lysosome increased	0 min	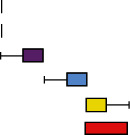
	5 min			5 min	
	10 min			10 min	
	30 min			30 min	
	60 min			60 min	
	120 min			120 min	
Chromatin pyknosis and border shift	0 min	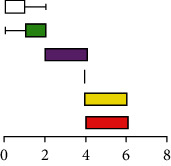	Nuclear shrinkage and fragmentation	0 min	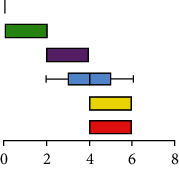
	5 min			5 min	
	10 min			10 min	
	30 min			30 min	
	60 min			60 min	
	120 min			120 min	

## Data Availability

The datasets supporting the conclusions of this article are included within the article.

## References

[1] Zhou X. (2005). Diagnostic pathology and its role in modern clinical medicine. *Journal of Medical Postgraduates*.

[2] Wu S., Liu X. (2018). The research progress of the phenomena and theory in autolysis. *Food Research and Development*.

[3] Babayan T. L., Bezrukov M. G. (2010). Autolysis in yeasts. *Acta Biotechnologica*.

[4] Zhao Z. (2004). *Forensic pathology*.

[5] Mcgavin L. L. M. D. (1972). Sequential postmortem changes in chicken liver at 4, 20, or 37 C. *Avian Diseases*.

[6] Tomita Y., Nihira M., Ohno Y., Sato S. (2004). Ultrastructural changes during in situ early postmortem autolysis in kidney, pancreas, liver, heart and skeletal muscle of rats. *Legal Medicine*.

[7] Zhou M. (2010). *Preliminary Study on Enzymatic Hydrolysis and Utilization of Engraulis Japonicus*.

[8] Kong M., Ji H. W., Zhang C. H. (2005). Autolysis conditions of tilapia byproduct proteins. *Journal of Zhanjiang Ocean University*.

[9] Huang X., Feng Y., Duan J. (2020). Antistarvation strategies of E. sinensis: regulatory networks under hepatopancreas consumption. *Oxidative Medicine and Cellular Longevity*.

[10] Xiong G., Yang Y. C., Feng Y. (2020). PCR diagnosis and histopathological damage of white spot disease of *Procambarus clarkii*. *Acta Agriculturae Universitatis Jiangxiensis*.

[11] Mejía L., Alvarez F., López M. (2003). Procambarus (Villalobosus) achilli (Decapoda, Cambaridae): a new species of crayfish from Mexico. *Crustaceana*.

[12] Baums C. G., Hermeyer K., Leimbach S. (2013). Establishment of a Model of *Streptococcus iniae* Meningoencephalitis in Nile Tilapia (*Oreochromis niloticus*). *Journal of Comparative Pathology*.

[13] Cao W., Zhang C., Hong P., Ji H. (2008). Response surface methodology for autolysis parameters optimization of shrimp head and amino acids released during autolysis. *Food Chemistry*.

[14] Morioka K., Fujii S. y., Itoh Y., Liu C., Obatake A. (2008). Recovery of amino acid from the protein in the head and viscera of frigate mackerel by autolysis. *Fisheries Science*.

[15] Senphan T., Benjakul S. (2012). Compositions and yield of lipids extracted from hepatopancreas of Pacific white shrimp (Litopenaeus vannamei) as affected by prior autolysis. *Food Chemistry*.

